# Utilization of the state led public private partnership program “*Chiranjeevi Yojana*” to promote facility births in Gujarat, India: a cross sectional community based study

**DOI:** 10.1186/s12913-016-1510-7

**Published:** 2016-07-15

**Authors:** Sandul Yasobant, Kranti Suresh Vora, Hemant Deepak Shewade, Kristi Sidney Annerstedt, Petros Isaakidis, Dileep V. Mavalankar, Nishith B. Dholakia, Ayesha De Costa

**Affiliations:** Indian Institute of Public Health-Gandhinagar, Sardar Patel Institute Campus, Drive-in-Road, Ahmedabad, Gujarat 380054 India; International Union Against Tuberculosis and Lung Disease (The Union), South East Asia Office, New Delhi, India; Karolinska Institutet, Solna, Sweden; Médecins Sans Frontières (MSF)/Doctors Without Borders, Mumbai, India; Department of Health & Family Welfare, Government of Gujarat, Gandhinagar, India

**Keywords:** Maternal mortality, Chiranjeevi Yojana, Demand side financing, Utilization, Institutional delivery, India

## Abstract

**Background:**

“Chiranjeevi Yojana (CY)”, a state-led large-scale demand-side financing scheme (DSF) under public-private partnership to increase institutional delivery, has been implemented across Gujarat state, India since 2005. The scheme aims to provide free institutional childbirth services in accredited private health facilities to women from socially disadvantaged groups (eligible women). These services are paid for by the state to the private facility with the intention of service being free to the user. This community-based study estimates CY uptake among eligible women and explores factors associated with non-utilization of the CY program.

**Methods:**

This was a community-based cross sectional survey of eligible women who gave birth between January and July 2013 in 142 selected villages of three districts in Gujarat. A structured questionnaire was administered by trained research assistant to collect information on socio-demographic details, pregnancy details, details of childbirth and out-of-pocket (OOP) expenses incurred. A multivariable inferential analysis was done to explore the factors associated with non-utilization of the CY program.

**Results:**

Out of 2,143 eligible women, 559 (26 %) gave birth under the CY program. A further 436(20 %) delivered at free public facilities, 713(33 %) at private facilities (OOP payment) and 435(20 %) at home. Eligible women who belonged to either scheduled tribe or poor [aOR = 3.1, 95 % CI:2.4 - 3.8] or having no formal education [aOR = 1.6, 95 % CI:1.1, 2.2] and who delivered by C-section [aOR = 2.1,95 % CI: 1.2, 3.8] had higher odds of not utilizing CY program. Of births at CY accredited facilities (*n* = 924), non-utilization was 40 % (*n* = 365) mostly because of lack of required official documentation that proved eligibility (72 % of eligible non-users). Women who utilized the CY program overall paid more than women who delivered in the free public facilities.

**Conclusion:**

Uptake of the CY among eligible women was low after almost a decade of implementation. Community level awareness programs are needed to increase participation among eligible women. OOP expense was incurred among who utilized CY program; this may be a factor associated with non-utilization in next pregnancy which needs to be studied. There is also a need to ensure financial protection of women who have C-section.

## Background

In the past two decades several low-and middle-income countries have implemented demand-side financial incentives (DSF) to improve healthcare service utilization and health-related behavior [1], specifically to address the maternal and child health related Millennium Development Goals (MDG-5) [2, 3]. MDG-5 and the Sustainable Development Goal (SDG-3.1) include the promotion of institutional deliveries as a strategy to reduce maternal mortality [4]. DSF programs incentivize a specified group to adapt a service or alter a behavior to improve health, education or help alleviate poverty. In maternal health, two of the most common types are voucher schemes where all or part of the cost of services are paid for, and cash transfer schemes where women are reimbursed for the costs of maternity services [2, 3, 5].

To increase the number of institutional deliveries, particularly among women from socially disadvantaged groups (i.e. women who lived below the poverty line or came from tribal castes–eligible women) in whom maternal deaths are more likely to occur, the Government of Gujarat, a large state of India, in 2005 implemented a large state-led DSF scheme in the form of a public-private-partnership (PPP), *Chiranjeevi Yojana* (CY). The CY (long life) was developed in response to an acute shortage of qualified obstetricians/gynecologists in the public health sector in Gujarat as most practiced in the private sector, where they received out of pocket payments for services rendered [6]. Under the CY program, eligible mothers could be beneficiaries by giving birth at an accredited private facility within the district, (led by a qualified obstetrician and free intra-partum care including emergency obstetric care) on presentation of a Government issued proof of poverty or tribal status. The government paid Indian Rupees (INR) 4000 per delivery (approximately $67) directly to the private facility irrespective of the type of birth i.e. vaginal or cesarean section (C-section). To date, about a million births have occurred under CY [7–9].

While overall institutional deliveries significantly increased from 58 % in 2004–06 to 95 % in 2010–12 [10–12], public sector deliveries rose only marginally from 24 % in 2001 to 29 % in 2010, indicating a large shift of childbirths occurring at home to the private sector [12]. Between 13 % and 16 % of all institutional deliveries from 2001 to 2010 occurred under the CY PPP [12, 13]. Since the implementation of the CY scheme, studies have shown it has been successful in increasing the geographic availability of free intra-partum care for eligible women [14]. However, considering that annually 570,625 births occur among eligible women and only 155,721 of these occur under CY, approximately 73 % of poor and tribal women did not utilize the free delivery service. This indicates there is scope for improvement in utilization of the scheme. Most of the previous research on CY has been from the health facility’ perspective [12, 15–17]. However, utilization among eligible women in the community needs to be studied in order to understand reasons for poor uptake of the CY program. This has not been reported in a previous community-based study on the CY published recently [7] or in older reports [9]. Though CY helped in saving $75 per delivery among those who utilized CY when compared to eligible women who delivered in a non-accredited private facility and paid fully out-of-pocket, out-of-pocket expenditures (OOPE) were still incurred among those who benefited from the program [9, 18].

This study attempts to document the factors associated with non-utilization of the CY program by eligible women who had recently given birth. Specific objectives were to i) determine the proportion of eligible women who utilized CY program; and ascertain among those who did not utilize the program the proportion delivering at home, Government facility, accredited private facility and non-accredited private facility, ii) determine the association between socio-demographic and pregnancy-related characteristics with non-utilization of CY program, iii) describe self-reported reasons for not availing CY benefit despite delivering at an accredited private facility, and iv) determine facility-wise OOPE with a focus on comparison of OOPE between those who delivered at Government facility and utilized CY after adjusting for type of delivery.

## Methods

### Study design

This was a community-based cross sectional study. This study was part of a large scale community-based Maternal Health India (MATIND) project aimed to study the CY program in the state of Gujarat.

### Study setting

#### Gujarat state

Gujarat state (population 60.4 million) is comprised of 26 districts (currently 33 districts), the average population of a district is two million. Districts are further divided into 10–20 blocks (sub-districts) of approximately 100,000 to 200,000 people. These districts have varying human development indices and different population compositions [19, 20]. The population is divided into socio-economic sub groups by caste. Government of India uses the terms ‘Schedule Caste’, ‘Tribe’ to denote these traditionally marginalized population subgroups. In addition the term ‘below poverty line’ (BPL) is also used to denote economically disadvantaged families. These two groups are recipients of official documentation from the government confirming tribal or BPL status. This documentation is used to avail special benefits under a broad program of positive affirmative action. We use the term ‘socially disadvantaged groups’ throughout the article which includes both schedule tribes (ST) and BPL.

### Study sites

**Fig. 1:**
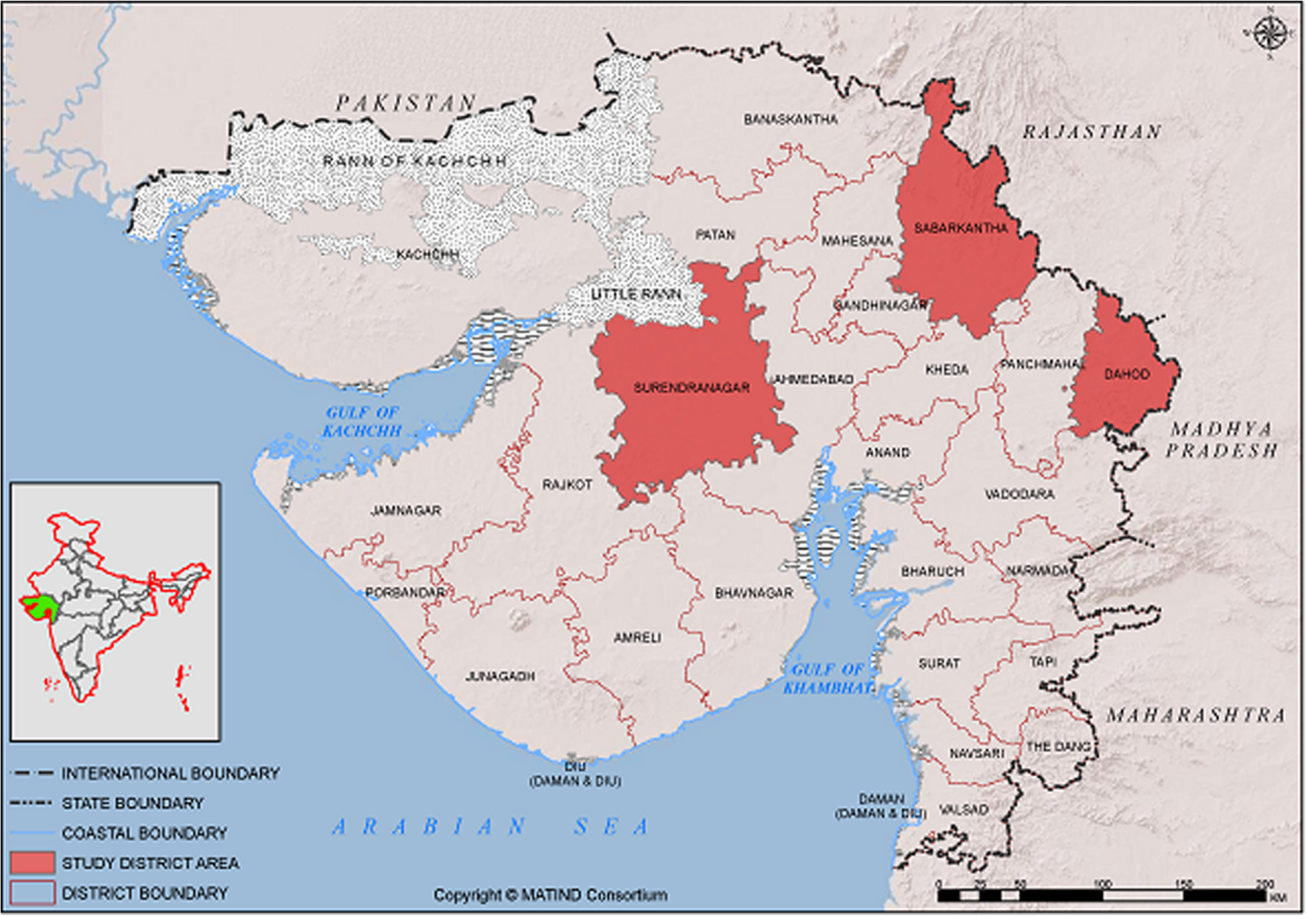
Map* of Gujarat (India) indicating the study districts (Dahod, Sabarkantha, Surendranagar). *The above map is a product of MATIND consortium and MATIND owns it’s copyright. Authors have written consent from MATIND consortium to use and adapt this map

Three heterogeneous districts from the western, central and eastern belts of the Gujarat state, Sabarkantha, Surendranagar and Dahod, (Fig. 1) were selected for the study. ‘The three districts were purposively selected, one each from high, medium and low human development index and different population compositions, i.e., varying proportions of tribes and populations living below the poverty line from diverse geographic areas. The detail indicators of these sampled districts are shown in Appendix Table.

### Study population and sampling

The study population included eligible women in the study districts who delivered between January and July 2013. As CY providers were not evenly distributed geographically throughout the district, blocks in each district were selected to represent areas where there were no, low (one or two providers) or high number (more than two per block) of CY providers. In brief, in each of the 3 districts, 3–5 blocks were selected purposively. A list of all the villages in these blocks was compiled using the criteria: village population more than 1,000 and less than 2,500, greater than 40 % BPL population and scattered all over the block. From the list, 142 villages were selected randomly to cover approximately 300,000 populations.

### Data collection and variables

Data collection was done between July and November 2014. Trained researcher visited the homes of eligible women who had given birth between January 2013 and July 2013 and administered a questionnaire. Local female village health volunteer helped researchers identify respondents. On an average, the administration of the questionnaire took 15 min.

#### Dependent variables

**Fig. 2:**
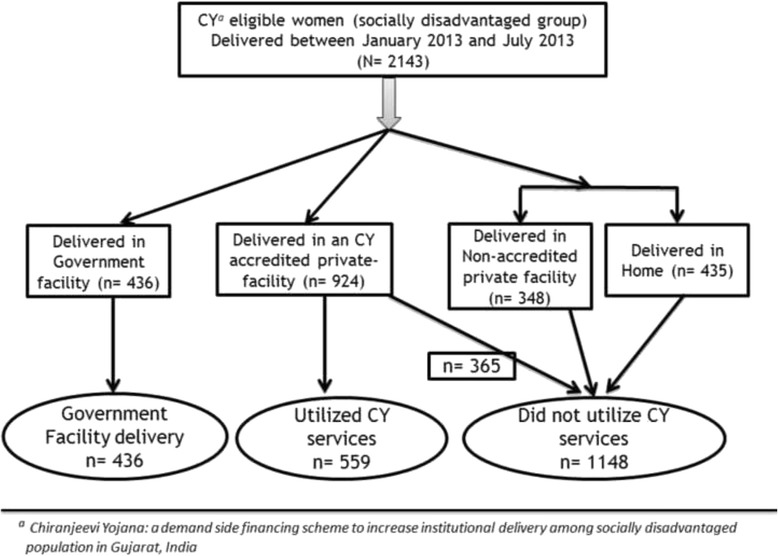
‘*Chiranjeevi Yojana* (CY)’^a^ utilization among eligible women who delivered between January-July 2013 in three districts of Gujarat, India (*N* = 2143)

For objective ii, the dependent variable was non-utilization of CY (Fig. 2) and for objective iv, it was OOPE. The operational definitions used were:

i. *Government facility delivery:* women who delivered at any government facility, which provided free institutional delivery care.
ii. *CY utilization* among eligible women was defined as delivery in an accredited facility and receipt of either completely free or partially subsidized intra-partum care under the CY.
iii. *CY non-utilization* comprised three different groups of women: (i) those who did not receive any benefit but delivered in a CY accredited facility, (ii) those who delivered in a non-accredited private facility or (iii) those who gave birth at home.
iv. OOPE: The total expense made by the woman/or her family including direct costs i.e. delivery care and indirect costs related to the birth i.e. treatment related other than delivery, transportation, food, and items purchased for the baby were expressed in USD (corrected for June 2015) after accounting for discounting for inflation between 2013 and 2015.

#### Independent variables

Socio-demographic characteristic collected were age, education categorized based on the number of years studied, earning status, religion, caste, and the standard of living index (SLI). The questionnaire included questions on household assets, living and sanitation conditions etc. from the National Family Health Survey and were used to create the SLI [21]. A score was calculated for each women based on pre-determined weights for 27 different components. The scores were subsequently divided into five quintiles (1^st^ being the poorest and the 5^th^ the wealthiest women in the survey) [22]. Pregnancy and childbirth associate variables collected were antenatal checkups defined as having a visit in the first trimester and at least three total antenatal visits during the pregnancy, self-reported pregnancy complications i.e. anemia, urinary tract infection, hypertension, parity, birth attendant, and type of delivery (i.e. vaginal or cesarean section).

### Data entry and analysis

Data from the interview scheduled were double-entered and validated in an online data management system i.e. Research Electronic Data Capture (REDCap) [23]. The database for this study was imported to EpiData analysis software v2.2.2.183 (EpiData Association, Odense, Denmark) for descriptive analysis and bivariate inferential analysis. STATA (version 12.1) was used for multivariable inferential analysis.

For objective i and iii, descriptive statistics (frequencies, proportions, median, and interquartile range) were used to summarize categorical and continuous variables. All independent variables and OOPE were described for the following categories: CY non-utilization, CY utilization and those delivering in Government facility.

Those who delivered in a Government facility were excluded from the analysis to study predictors associated with CY non-utilization. Variables with univariate *p*-value <0.2 were included in the binomial logistic regression model (forward LR method). Adjusted odds ratio (aOR) was used to describe the association between CY non-utilization (outcome) and socio-demographic/pregnancy related characteristics (exposure). Prior to building the model, we checked for collinearity between predictor variables and if collinear, then the variables were excluded from the model.

Among women who should have received free delivery services under either the CY or in government facilities, we compared out-of-pocket expenditures. Beta coefficient (linear regression–forward method) was used to summarize the association between log_10_OOPE (outcome) and CY utilization (exposure) after adjusting for type of delivery and other variables. Log_10_ transformation was performed on the outcome variable (OOPE) as it was not normally distributed. 95 % confidence interval (CI) was used to infer the above summary statistics.

## Results

### Study sample characteristics and CY utilization

As shown in Fig. 2, among the 2,143 eligible women, 559 (26 %) were CY beneficiaries. A further 365 (17 %) gave birth at CY accredited facilities but did not benefit from the program, 436 (20 %) delivered at Government facilities, 348 (17 %) at non-accredited CY private facilities and 435 (20 %) at home. The socio demographic characteristics of the eligible women are shown in Table 1. There was a higher proportion of mothers with no formal education among those giving birth at home (62 %) and delivering in Government facilities (53 %), compared to those who utilized CY (32 %), delivered in CY accredited but did not utilize CY (40 %) and delivered in non-accredited private (31 %) facilities. As shown, the women in the survey were mostly multiparous with a majority having at least one ANC check-up. A higher proportion of primiparous mothers utilized CY (44 %) when compared to primiparous women who delivered at home (25 %). Similarly as seen in Table 2, a higher proportion of women with no ANC check-ups were found among those who gave birth at home (20 %) compared those who utilized CY (9 %). There was a lower proportion of intra-natal (INC) complication (3 %), ANC complication (4 %) and C-sections (3 %) among mothers who utilized CY when compared to deliveries that occurred in CY accredited but did not utilize CY and non CY accredited facilities.

**Table 1:** Socio-demographic characteristics and ‘Chiranjeevi Yojana (CY)’^a^ utilization among eligible women^b^ in three districts^c^ of Gujarat, India (Jan-July 2013) (*N*=2143)

Characteristic		Utilized Government facility^d^	Utilized CY^d^	Did not utilize CY^d^		
				Private CY accredited	Private non-accredited	Home delivery
		N (%)	N (%)	N (%)	N (%)	N (%)
Total		436 (100)	559 (100)	365 (100)	348 (100)	435 (100)
Eligibility criteria^e^						
	BPL^f^ or ST^g^	348 (80)	324 (58)	271 (74)	312 (90)	364 (84)
	BPL & ST	88 (20)	235 (42)	94 (26)	36 (10	71 (16)
Age						
	≤ 25	277 (64)	355 (63)	251 (69)	245 (70)	230 (53)
	>25	159 (36)	204 (37)	114 (31)	103 (30)	205 (47)
Education						
	No Education	232 (53)	182 (32)	144 (40)	109 (31)	270 (62)
	Primary Education	45 (10)	49 (9)	34 (9)	47 (14)	39 (9)
	Secondary Education	134 (31)	238 (43)	132 (36)	143 (41)	103 (24)
	≥ Higher					
	Secondary	25 (6)	90 (16)	55 (15)	49 (14)	23 (5)
Earning status						
	No	248 (57)	367 (66)	219 (60)	222 (64)	289 (66)
	Yes	188 (43)	192 (34)	146 (40)	126 (36)	146 (34)
Religion						
	Hindu	432 (98)	549 (98)	362 (99)	345 (99)	433 (100)
	Muslim	2 (1)	4 (1)	3 (1)	3 (1)	1 (0)
	Christian	2 (1)	6 (1)	0	0	1 (0)
Standard of living index						
	1^st^ quintile	87 (20)	132 (24)	57 (16)	38 (11)	114 (26)
	2^nd^ quintile	93 (21)	119 (21)	61 (17)	51 (15)	104 (24)
	3^rd^ quintile	92 (21)	118 (21)	74 (20)	60 (17)	84 (19)
	4^th^ quintile	94 (22)	100 (18)	88 (24)	72 (21)	74 (17)
	5^th^ quintile	70 (16)	90 (16)	85 (23)	127 (36)	59 (14)

^a^demand side financing scheme to increase institutional delivery among socially disadvantaged population; ^b^women belonging to socially disadvantaged population; ^c^Dahod, Sabarkantha, Surendranagar; ^d^column percentage; ^e^socially disadvantaged groups which includes both schedule tribes and below poverty line; ^f^Below poverty line; ^g^Schedule tribes

**Table 2:** Pregnancy related characteristics and ‘Chiranjeevi Yojana (CY)’^a^ utilization among eligible women^b^ in three districts^c^ of Gujarat, India (Jan-July 2013) (*N*=2143)

Characteristic		Utilized Government facility^d^	Utilized CY^d^	Did not utilize CY^d^		
				Private CY accredited	Private non-accredited	Home delivery
		N (%)	N (%)	N (%)	N (%)	N (%)
Total		436 (100)	559 (100)	365 (100)	348 (100)	435 (100)
Parity						
	Primipara	157 (36)	247 (44)	180 (49)	164 (47)	108 (25)
	Multipara	279 (64)	312 (56)	185 (51)	184 (53)	327 (75)
Antenatal visits						
	No	43 (10)	50 (9)	33 (9)	7 (2)	85 (20)
	<3 visits	92 (21)	86 (15)	62 (17)	49 (14)	108 (25)
	≥ 3 visits	301 (69)	423 (76)	270 (74)	292 (84)	242 (55)
Antenatal complication						
	No	417 (96)	538 (96)	335 (92)	318 (91)	420 (97)
	Yes	19 (4)	21 (4)	30 (8)	30 (9)	15 (3)
Intranatal complication						
	No	425 (98)	542 (97)	330 (90)	317 (91)	434 (100)
	Yes	11 (2)	17 (3)	35 (10)	31 (9)	1 (0)
Delivery type						
	Vaginal	420 (96)	544 (97)	329 (90)	315 (90)	435 (100)
	C-Section	16 (4)	15 (3)	36 (10)	33 (10)	0
Birth attendant						
	Nurses	304 (70)	126 (23)	72 (20)	68 (20)	6 (1)
	Unqualified Staff at facility	5 (1)	157 (28)	66 (18)	21 (6)	5 (1)
	Doctors	75 (17)	17 (3)	17 (5)	33 (9)	18 (4)
	Gynecologists	52 (12)	259 (46)	210 (55)	226 (65)	0
	Relative/TBA^e^	0	0	0	0	406 (94)

^a^demand side financing scheme to increase institutional delivery among socially disadvantaged population; ^b^women belonging to socially disadvantaged population; ^c^Dahod, Sabarkantha, Surendranagar; ^d^column percentage, ^e^Traditional Birth Attendant

### Determinants of CY non-utilization

Eligible women who belonged to scheduled tribe or BPL [aOR = 3.1, 95%CI:2.4 - 3.8] or and having no formal education [OR = 1.6, 95%CI:1.1,2.2] had higher odds of not utilizing CY scheme. Women who delivered by C-section had twice the odds [aOR = 2.1,95 % CI: 1.2, 3.8] of not utilizing CY. Standard of living, parity, ANC visits and ANC complications had high collinearity with delivery type and were therefore not included in the model (Table 3).

**Table 3:** Binomial logistic regression^a^ for factors associated with non-utilization of ‘Chiranjeevi Yojana (CY)’ ^b^ among eligible women^c^ in three districts^d^ of Gujarat, India (Jan-July 2013) (*N*=1707)^e^

Variable^f^		Crude OR (95 % CI)	Adjusted OR (95 % CI) ^g^
Socio-demographic characteristics			
Eligibility criteria ^h^			
	BPL or ST	3.4 (2.7, 4.3)	**3.1 (2.4, 3.8)**
	BPL and ST	Reference	Reference
Age			
	18–25	Reference	Reference
	>25	1.0 (0.8, 1.2)	0.98 (0.8, 1.2)
Education			
	No Education	2.0 (1.5, 2.8)	**1.6 (1.1, 2.2)**
	Pri. Education	1.7 (1,1, 2.7)	1.2 (0.8, 1.9)
	Sec. Education	1.1 (0.8, 1.5)	1.0 (0.7, 1.4)
	≥ Higher Sec.	Reference	Reference
Standard of living index			
	1^st^ quintile	Reference	-
	2^nd^ quintile	1.2 (0.8, 1.6)	-
	3^rd^ quintile	1.2 (0.9, 1.6)	-
	4^th^ quintile	1.5 (1.1, 2.0)	-
	5^th^ quintile	1.5 (1.1, 2.0)	-
Pregnancy related characteristics			
Parity			
	Primipara	Reference	-
	Multipara	1.2 (0.99, 1.5)	-
Antenatal visits			
	No	1.3 (0.9, 1.9)	-
	<3 visits	1.3 (1.02, 1.8)	-
	≥ 3 visits	Reference	-
Antenatal complication			
	No	Reference	-
	Yes	1.8 (1.1, 2.9)	-
Delivery type			
	Vaginal	Reference	Reference
	C-Section	2.3 (1.3, 4.1)	**2.1 (1.2, 3.8)**

^a^Forward LR method with CY non-utilization as outcome; ^b^demand side financing scheme to increase institutional delivery among socially disadvantaged population; ^c^women belonging to socially disadvantaged population; ^d^Dahod, Sabarkantha, Surendranagar; ^e^Women who delivered in Government facility were excluded; ^f^variables with bivariate *p*<0.2 shown in table, ^g^Standard of living, parity, ANC visits and ANC complications had high collinearity with delivery type and were therefore not included in the model, ^h^sociallydisadvantaged groups which includes both schedule tribes and below poverty line

Model chi square: 134.6, df: 12, *p*<0.001; Pseudo Rsquare: 0.06; Hosmer and Lemeshow Chi square: 8.15, df: 8, *p*=0.419

OR mentioned as bold are significant values in the model

### Reasons for CY non-utilization in a CY facility

Non-utilization of CY among eligible mothers delivering at CY accredited facility was 40 % (365/924). The reasons included: (i) no documentation i.e. what is essential in order to get the benefit at the time of delivery (*n* = 262, 72 %), (ii) mother belonged to a different district (*n* = 76, 21 %), and (iii) lack of CY program awareness (*n* = 36, 10 %).

### CY utilization and OOPE

Median OOPE stratified by place of delivery is described in Table 4. Overall, the median OOPE those who did not utilize CY at accredited and non-accredited private facility was $75 and $90, respectively. Median OOPE among those who utilized CY and those delivering at a Government facility was $12 and $3, respectively. Median OOPE among home delivery mothers was found to be $5. CY utilization resulted in significantly higher OOPE compared to women who delivered in a Government facility after adjusting for type of delivery (Table 5).

**Table 4:** Median delivery related out of pocket expenditure^a^ and ‘Chiranjeevi Yojana (CY)’^b^ utilization among eligible women^c^ who delivered between Jan-Jul 2013 in three districts^d^ of Gujarat, India (*N*=2143)

Characteristic	Utilized Government facility	Utilized CY	Did not utilize CY		
			Private CY accredited	Private non-accredited	Home delivery
Delivery expenses	0 (0–0)	0 (0–8.3)	58.3 (41.7–83.3)	66.7 (50–100)	3.3 (0–9.6)
Other treatment expenses	0 (0–0)	0 (0–0)	0 (0–0)	0 (0–2.1)	0 (0–0)
Food expense	0 (0–0)	0 (0–0)	0 (0–0)	0 (0–3.3)	0 (0–0)
Transportation expense	0 (0–5)	6.7 (1.7–8.3)	8.3 (3.3–13.3)	6.7 (3.3–11.7)	0 (0–0)
Baby expenses	0 (0–0)	0 (0–0)	0 (0–0)	0 (0–1.7)	0 (0–0)
Total [median, (IQR)]	3.3 (0–10)	11.7 (5–23.3)	75 (53.3–103.3)	90 (60–131.7)	5 (0–10)

^a^USD (corrected for June 2015) after accounting for discounting, ^b^demand side financing scheme ‘Chiranjeevi Yojana (CY)’ to increase institutional delivery among socially disadvantaged population; ^c^women belonging to socially disadvantaged population; ^d^Dahod, Sabarkantha, Surendranagar

**Table 5:** Factors associated with out-of pocket expenditure^a^ among women eligible^b^ for CY^c^ who delivered between Jan-Jul 2013 in three districts^d^ of Gujarat, India (*N*=995)

Variable^e^		β coefficient	0.95 CI	*p*-value^f^
Place of delivery				
	CY utilizer	0.21	0.15, 0.27	<0.001
	Government facility	Reference		
Type of delivery				
	C-section	0.80	0.66, 0.95	
	Vaginal	Reference		<0.001
Eligibility criteria ^g^				
	BPL or ST	0.07	0.01,0.13	
	BPL and ST	Reference		0.02
Constant		0.67	[0.47, 0.86]	<0.001

^a^USD (corrected for June 2015) after accounting for discounting followed by log transformation; ^b^ women belonging to socially disadvantaged population; ^c^demand side financing scheme ‘Chiranjeevi Yojana (CY)’ to increase institutional delivery among socially disadvantaged population; ^d^ Dahod, Sabarkantha, Surendranagar; ^e^Variables added to linear regression model (forward method) were place of delivery, delivery type, eligibility criteria, age group and education status: latter two were not included by the model; ^f^ Standard of living, parity, ANC visits and ANC complications had high collinearity with delivery type and were therefore not included in the model; ^g^socially disadvantaged groups which includes both schedule tribes and below poverty line

Model F stat: 54.5; *p*<0.001

## Discussion

This study highlighted that of eligible mothers, after excluding government delivery; more than two-thirds did not utilize CY program benefits. Although 20 % of the women delivered in public sector facilities, there was scope for shifting those from delivering at home or at a private facility (including an accredited facility where women faced barriers) into CY. Though the non-utilization group is heterogeneous (women who gave birth at home, women who delivered in non-accredited CY private facilities and women who delivered in a CY accredited facility but did not receive the benefit), we need different strategies for different group. Interestingly, about two-fifths of eligible women who delivered at a CY facility did not receive the benefit of a free delivery. In addition, those who utilized CY incurred higher out-of-pocket expenses when compared to women who delivered in a Government facility.

### CY non-utilization & child birth at home

First and foremost is the reason for non-utilization and most important group belong to women who gave birth at home. Though home deliveries were found only in 8 % of all deliveries in the state [24], this study found that about 20 % of these socially disadvantaged mothers gave child birth at home; this indicates that home based child birth is more concentrated in these socially disadvantaged group. These mothers were poor, young, having no formal education, multiparous and had no/<3 ANC visits, which is similar in line of previous predictor-based study on home delivery [25]. This group may be targeted early on in the antenatal period with awareness generation programs for non-literate mothers about the scheme and the documentation requirement [26]. Specific efforts are needed to help these mothers obtain the required documentation which could be facilitated and monitored by a community health worker (the Accredited Social Health Activists (ASHA)). The same awareness programs and efforts by ASHA should target home deliveries.

### CY non-utilization & child birth at non-accredited private facilities

The second group targets women who delivered in non- accredited CY private facilities. Eligible women belonging to higher quintiles of the standard of living index (relatively better off among vulnerable women) were also more likely to not utilize the scheme. Women with higher education and women who had adequate ANC visits gave birth in non-accredited CY private facilities: relatively well-off women among the eligible population may more choice for place of delivery and they choose to have child birth in a private facility.

### CY non-utilization & child birth at CY accredited private facilities

Despite reaching to a CY facility there are group of women (about 40 %) who become did not utilize CY. In our previous facility based study [18], about two-third of eligible women who reached a CY facility did not utilize CY, which is higher than this report. Similar to previous study [18] most common reason given for non-utilization by the women was lack of proper documentation and being unaware of the CY scheme itself or of paperwork necessary to become a beneficiary. This was also highlighted by Bhimani et al.; most of the participants who were actually the beneficiaries of the scheme were unaware of ‘Chiranjeevi Scheme’ and authors recommended that Information Education & Communication (IEC) activities with emphasis on Government programs focusing on maternal and child health should be strengthened [27]. Women who had a C-section delivery were more likely to not utilize the program in our study. Though we did not explore why this was the case, a previous study in our setting suggested that private accredited facilities would sometimes shift potential cesarean section (C-Section) births out of the CY program because of the bundled financial reimbursement package which did not specifically incentivize C-Section [16].

### CY utilization & out-of-pocket expenditures

Interestingly, in comparison, women who gave birth at home paid some out of pocket money ($5) for services rendered by the birth attendant, which was even higher than from giving birth at government facilities ($3.3). Similar to previous studies [9, 18] this study found that women receiving the CY benefit had some delivery related expenditure ($11.70) and it was significantly less than those who did not utilize CY scheme and delivered in a private facility ($75–$90). Sidney et al. [18] also highlighted that; CY beneficiaries experienced a substantially subsidized childbirth compared to women who delivered in non-accredited private facilities, but often not completely free childbirth services as envisaged by the program. Our study found that OOPE among CY utilization was significantly higher than for those giving birth at a Government facility. OOPE among mothers who utilized CY are expected to be either zero or at best similar to OOPE in a Government facility. While women, who utilized the CY PPP, did not incur any expenses towards services in the facility, they still had other expenditures particularly travel and therefore overall paid more than women who delivered in the public sector. Although this was not explored, one could postulate that OOPE despite CY utilization could be a factor resulting in non-utilization in next delivery. A study by Vora et al. found that women travelled longer distance to avail benefits from CY services [14]. This distance involved is likely to contribute to expenses related to transportation which CY beneficiaries incurred, but not women who give birth in closer government facilities.

### Methodological consideration

The main strength of this study is that this is a large primary community based survey that reported for the first time on the uptake of the CY program based on empirical data. One of the limitations of this study was that we did not explore the reasons behind child birth at home or in government or non-accredited private facilities. CY non-utilization was a heterogeneous group; this might have affected the estimates in our analysis. In addition, assessment of eligibility for CY scheme by the investigators was done at the time of survey. We believe that the study finding are representative, acknowledging the fact that our sampling method not being random might limit the extrapolation of the study finding to the population of CY eligible mothers.

### Policy implications

First, ensuring that a woman has appropriate eligibility documentation at the time of delivery is important to gain access to the CY program. A convergence approach can be considered where all accredited health facilities have access to the available list of eligible women/families in their district. This will allow more women to become beneficiaries automatically if they are in the list at each facility rather than placing the onus of providing appropriate documentation on the women/family. There are two groups that need focus in terms of increasing uptake of the program, one are those who delivered outside a CY facility (women who delivered at home received no care and women who delivered in private facilities, both of whom had OOPE) and those who managed to get to a CY facility but didn’t get the become CY beneficiaries. Community awareness needs to be strengthened to reach both these groups to improve uptake of the CY PPP.

Second, more effective monitoring of private partners is necessary to ensure that there is no ‘cream-skimming’ of cases so that women with complications are not excluded from CY. Third, women who utilized CY still face some financial access barriers in the form of indirect costs, particularly transport which could be a deterrent to utilization by poor women. Further research is required to determine whether OOPE in utilization in current delivery results in non-utilization in the next birth.

## Conclusion

Our study showed that almost a decade of implementation, the uptake of the Chiranjeevi Yojana among eligible women is still low. There is a need for community level awareness programs to increase participation among eligible women. The out-of-pocket expenses to cover indirect costs associated with CY utilization among socially disadvantaged group needs to be looked into. There is also a need to put in place monitoring mechanisms to ensure women with complications are not excluded from the CY program.

## Abbreviations

ANC, antenatal care; aOR, adjusted odds ratio; ASHA, accredited social health activists; BPL, below poverty line; CI, confidence interval; C-section, cesarean section; CY, Chiranjeevi Yojana; DSF, demand-side financial incentives; IEC, information education & communication; INR, Indian Rupees; MATIND, maternal health India; MDG, millennium development goals; OOPE, out-of-pocket expenditures; PPP, public-private-partnership; REDCap, research electronic data capture; SDG, sustainable development goal; SLI, standard of living index; ST, schedule tribe

## Appendix

**Table 6:** Demographic characteristics of MATIND ^a^ study districts of Gujarat, India

Indicators ^b^	Dahod	Sabarkantha	Surendranagar
Populations (in millions)	2.1	2.4	1.7
Rural population (%)	91	85	72
BPL^c^ population (%)	72	33	47
ST^d^ population (%)	73	22	01
Literacy rate (%)	59	65	62
Total private providers (number)	26	83	38
CY^*e*^-accredited private providers (number)	8	23	21

^a^Maternal Health India Project, ^b^Size, Growth Rate and distribution of Population-Provisional Population Totals: Census of India-2011 ^c^Population living in Below Poverty Line; ^d^Population belongs to Social Tribe; ^e^demand side financing scheme ‘Chiranjeevi Yojana (CY)
